# In-Vehicle Visible Light Communications Data Transmission System Using Optical Fiber Distributed Light: Implementation and Experimental Evaluation

**DOI:** 10.3390/s22186738

**Published:** 2022-09-06

**Authors:** Cătălin Beguni, Alin-Mihai Căilean, Sebastian-Andrei Avătămăniței, Eduard Zadobrischi, Raul Stoler, Mihai Dimian, Valentin Popa, Bastien Béchadergue, Luc Chassagne

**Affiliations:** 1 Integrated Center for Research, Development and Innovation in Advanced Materials, Nanotechnologies, and Distributed Systems for Fabrication and Control, Stefan cel Mare University of Suceava, 720229 Suceava, Romania; 2 Department of Computers, Electronics and Automation, Stefan cel Mare University of Suceava, 720229 Suceava, Romania; 3 Laboratoire d’Ingénierie des Systèmes de Versailles, Paris-Saclay University, 78140 Vélizy-Villacoublay, France; 4 Department of Computer Science, Technical University of Cluj-Napoca, 400027 Cluj-Napoca, Romania

**Keywords:** in-car communications, in-vehicle communications, optical communications, optical fiber distributed light, traffic safety, visible light communication, wireless optical communications

## Abstract

Visible light communications emerges as a promising wireless communication technology that has been found suitable for numerous indoor and outdoor applications. In this article, a new in-vehicle VLC system is designed, implemented, and experimentally evaluated. The purpose of this new system is to provide car passengers with optical wireless communications. The proposed system consists of a VLC emitter integrated into the vehicle’s ambient lighting system and a mobile VLC receiver. Unlike any previous works, this article proposes a VLC emitter in which the light from a 3 W LED is distributed on a 2 square meter surface using 500 optical fibers whose main purpose is a decorative one. The proposed prototype has been implemented on a car and evaluated in relevant working conditions. The experimental evaluation of the proposed system has demonstrated the viability of the proposed concept and showed a data rate of 250 kb/s while providing a BER lower than 10^−7^. As far as we know, the proposed concept is totally new in the VLC literature, opening a new area of utilization for VLC technology: using VLC with optical fiber distributed light.

## 1. Introduction

Visible light communications (VLC) is a branch of optical wireless communications (OWC) that managed to gather a lot of attention from the research community but also from the general public [1,2]. VLC is a remarkable technology that assumes the dual use of solid-state lighting sources, namely, lighting and data communications. In turn, this approach ensures several important benefits that provide it with huge development potential. Thus, different from any other wireless communication technologies, VLC is developing on top of the pre-existing lighting infrastructure, providing the opportunity for fast and cost-efficient development and deployment. Additionally, the basic principles of VLC assume that the data are carried by the optical carrier, with no additional power consumption being required. Corroborated with the very low power consumption associated with LEDs, energy efficiency becomes extremely important in the context in which human society is beginning to be extremely interested in developing green technologies and in reducing energy consumption [3].

The high potential of VLC technology has been also confirmed by its standardization by the IEEE organization. Thus, soon after the first VLC prototypes became available, VLC technology was standardized by the IEEE 802.15.7 standard in 2011 [4,5,6], and several other standard updates became available later [7,8,9]. Following this favorable trend, VLC technology began developing, and thus, numerous applications have been identified. Hence, right from the beginning, the main application of VLC has been providing extremely high data rate wireless connections, being suitable for broadband internet [10,11,12]. In this area, VLC technology proved capable of ensuring tens of gigabits per second data rates [10], with connections above 100 Gb/s being envisioned [11]. Consequently, VLC began being considered a very promising candidate in 5G–6G applications [12,13,14,15,16]. In this area, VLC is extremely suitable due to the spatial reuse capabilities that recommend it for the small cell concept. In turn, 5G–6G technologies can benefit from higher-than-ever data rates, extremely low latencies (<1 ms), and wide coverage.

The wide area distribution of LED light sources, together with the VLC energy harvesting [17,18] and energy transfer capabilities [19,20], also opens the possibility of being used in Internet of Things [21,22] applications. Additionally, the current Industry 4.0 transition forces the integration of wireless communication technologies into more and more industrial applications. Here, VLC can provide simple deployment, cost-efficiency, improved flexibility, and high scalability, being a suitable candidate for this area as well [23,24]. In a rather similar but more specific area, VLC can be also used for robot control, localization, and guidance [25,26]. In such applications, VLC, together with visible light positioning (VLP) [27,28], can be used to provide autonomous robots with commands while simultaneously enabling their centimeter precision positioning accuracy, which in turn, can significantly improve robots’ performances.

Another extremely popular use case for VLC technology is its use in communication-based vehicle safety applications [29,30,31,32,33,34,35]. In this field, VLC prototypes proved their merits [32], being capable of providing adequate noise resilience [31], communication ranges of almost 200 m [33], and latencies that satisfy the requirements of automotive communication applications [34,35].

The fact that unlike other wireless communication technology, VLC is totally safe for the human body and also for highly sensitive electronic equipment, recommends its use in RF-restricted areas. Consequently, VLC technology can be used in medical applications [36,37], in airplanes [38,39], or in locations where RF-based technologies might generate utilization problems (i.e., nuclear plants, oil platforms, underground mines) [40]. Last but not least, a very important use case for VLC technology is underwater communications [41,42]. In such applications, VLC technology offers unique benefits such as high bandwidth and high data rates while being green, clean, and safe [42]. In these conditions, some consider that VLC has a high potential in short- to medium-range underwater communication applications.

**Figure 1:**
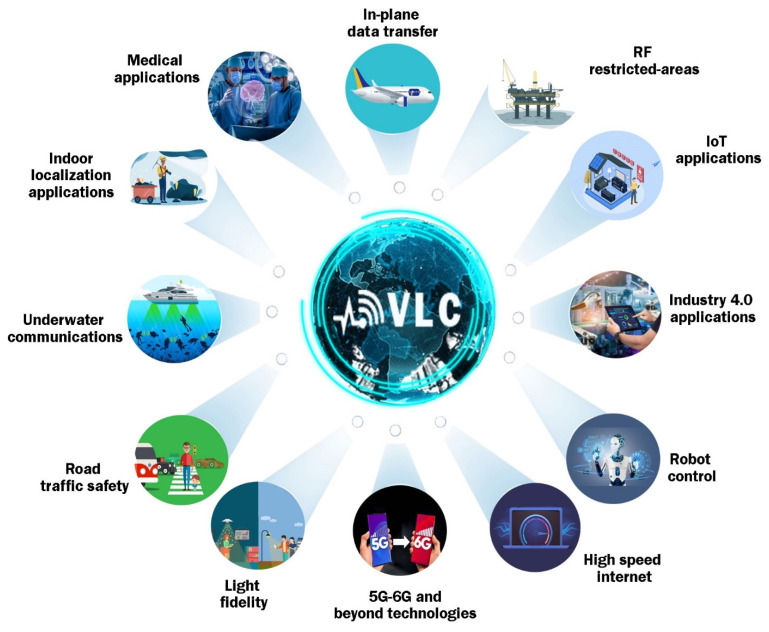
Summary of some of the most representative visible light communications utilization scenarios and applications.

As one can see, in the current context in which human society is expressing an increasing demand for high data rate wireless communication technologies but also for green and safe technologies, VLC has an extremely high potential for being used in an extremely vast area of applications, as summarized in Figure 1. Additionally, this potential is being exploited by an increasing number of research groups that are focused on its development, in order to find new applications and overcome some of its well-known issues, such as increasing the immunity to weather conditions, maximum communication distances, and data rates.

In the above-presented context, this article introduces a new VLC application: the use of VLC technology for in-vehicle communications purposes with the help of optical fiber distributed light. Although the article is hardware-oriented rather than application orientated or user-centric, the experimental results demonstrate the fact that cars’ ambient lighting systems can be successfully used for VLC purposes, with the help of optical fibers instead of copper wires and LEDs. Toward this goal, this article presents the aspects related to the design, implementation, and experimental evaluation of a VLC system designed to provide optical wireless communications coverage for the occupants of a vehicle. Therefore, a VLC emitter was developed and integrated into a car, with an ambient lighting system based on optical fibers. For VLC data reception, a VLC receiver prototype has been designed and implemented. The experimental results demonstrate the viability of the proposed concept, as it has been shown that occupants of the vehicle are able to adequately receive data in normal car-sharing conditions. Nevertheless, as this article is presenting an innovative concept, together with a novel demonstrative prototype and not a new commercial system, the system performance has not reached its maximum potential. As far as we know, this is the first article reporting an in-vehicle data VLC transmission using the car’s ambient lighting system, and most importantly, this is the first article reporting the implementation of a VLC system in which visible light containing data is being guided by optical fibers before reaching free space.

The rest of this article is organized as follows. Section 2 presents the aspects related to the hardware design and implementation of the VLC prototype, consisting of an in-vehicle VLC emitter and a mobile VLC receiver. Section 3 approaches the issues related to the intensive simulation and experimental evaluation of the VLC system. Section 4 provides a debate concerning the results of this article and emphasizes the importance of this work, and Section 5 delivers the conclusions of this article.

## 2. Design and Implementation of the In-Vehicle Visible Light Communications Data Transmission System

### 2.1. Motivation and Guidelines

Modern society implies a lot of mobility but also assumes that, unlike in previous times, users still desire to remain connected with their activities or to work on their activities even while traveling from one place to another. This assumes the presence of a reliable, trustworthy, and ubiquitous wireless communication technology or group of technologies. Additionally, users require high data rate connections and reduced latencies. The protection of the data is also of significant importance.

**Figure 2:**
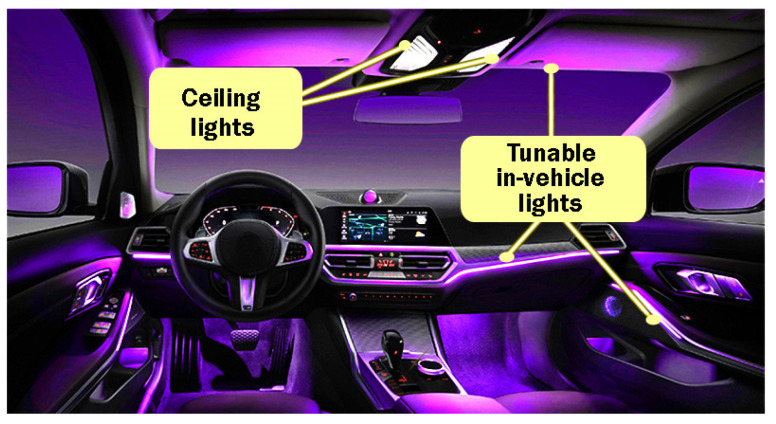
Tunable in-vehicle ambient lighting system.

Another aspect associated with modern people’s habits is related to their personalized items. Nowadays, people like and are willing to pay for unique or limited-edition options. In the automotive area, this translates into cars that have unique looks and personalized features, such as motor tuning, improved exterior design, and different kinds of interior features. When discussing interior car improvements, an important feature is provided by the ambient lighting system. Thus, current manufacturers integrate their series vehicles with tunable lighting functions that enable the user to personalize the interior ambiance of the car. These features provide a great aesthetic effect and enable car owners to illuminate the interior of the car in accordance with their preferences. Thus, with the help of RGB LEDs, the entire spectrum of colors can be generated according to users’ choices. Figure 2 illustrates such an example.

In light of the above-mentioned, this article proposes the use of adjustable ambient lighting systems for VLC data transmission, having not only the convenience but also the safety of travel in mind. Indeed, the VLC alternative to the RF-based communication can be of real help in critical road traffic scenarios, such as traffic congestion or long tunnel passage, when interference or poor reception can humper a traditional wireless connection, impeding, for example, some necessary occupations for the kids in the car. Consequently, the next subsections debate the aspects related to the practical design and implementation of the in-vehicle VLC system.

### 2.2. Hardware Design and Implementation of the In-Vehicle Visible Light Communications Emitter

Although the VLC emitter is not the central element in the design of a VLC system, this part is very important for users. From this perspective, the IEEE 802.15.7 standard for optical communications using visible light clearly stipulates that the VLC function is a complementary function of the lighting device and that the data transmission ability should not affect, influence, or limit the primary lighting function. Thus, in order to comply with these regulations, VLC systems must not induce any noticeable or perceivable flickering and should include light dimming functions if the original application requires it. For this purpose, but also to reduce the implementation cost, optical fibers were chosen to redirect the light within the car. Thus, instead of using 500 micro-LEDs, the VLC emitter is using a 3 W RGB LED and 500 optical fibers that carry the LED light to 500 points of the passenger compartment. This solution generated a very user-attractive design while offering a lower cost with respect to the case when multiple LEDs would have been chosen.

**Figure 3:**
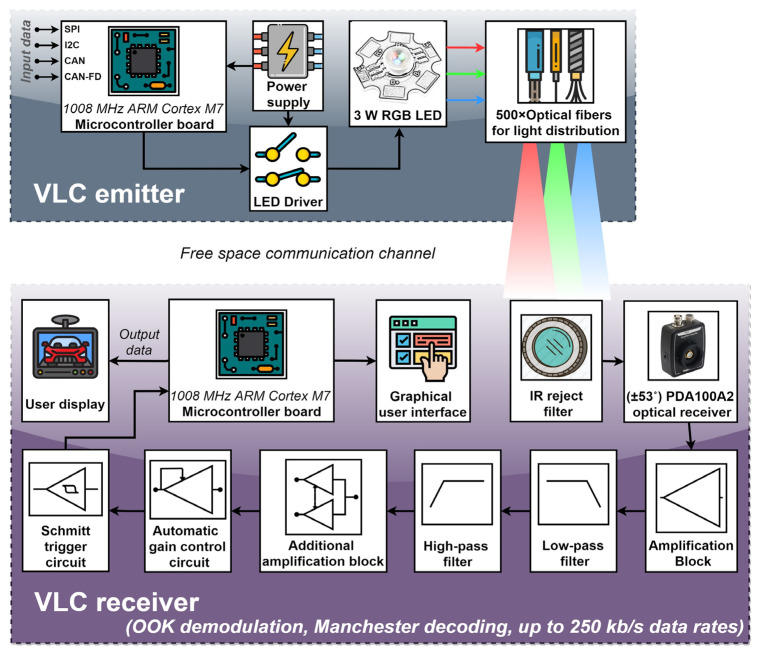
Schematic representation of the proposed in-vehicle visible light communications system.

**Figure 4:**
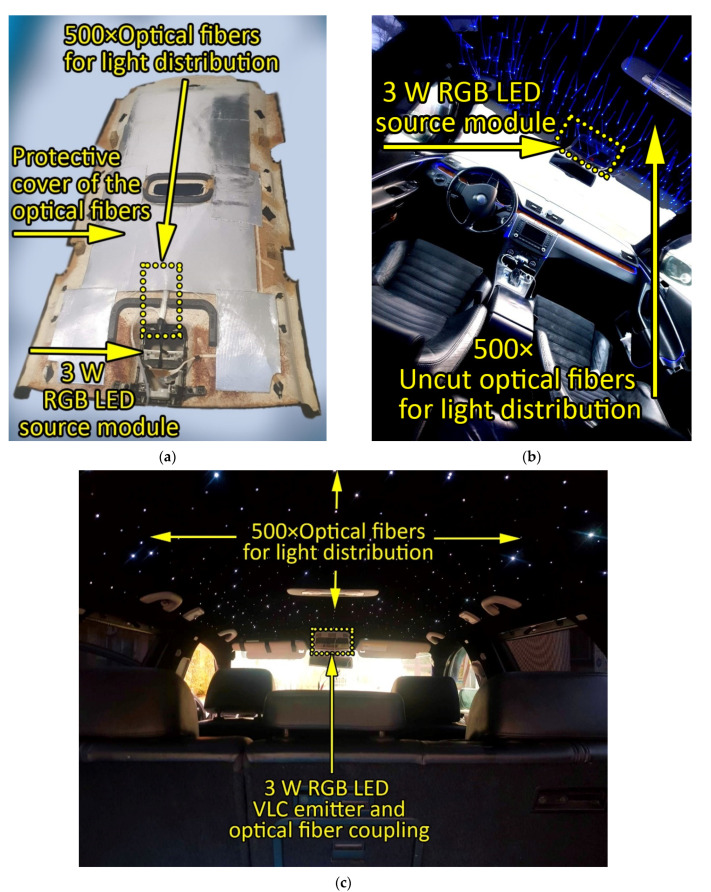
Hardware implementation of the ambient light in-vehicle visible light communications emitter: (**a**) installation of the optical fiber within the vehicle rooftop; (**b**) adjustment of the optical fiber within the vehicle rooftop; (**c**) final in-vehicle optical wireless data transmission system based on optical fiber distributed light.

Figure 3 presents the diagram of the proposed VLC system, englobing the VLC emitter and the VLC receiver. As one can see, a 1008 MHz ARM Cortex M7 Microcontroller board is the central element of the in-vehicle VLC emitter. The microcontroller deals with the data transmission function, and thus, it transforms the data to send into a bit stream. Therefore, the microcontroller is responsible for data processing, data encoding, data modulation, and data frame building. Due to its simplicity and decent performance, the VLC emitter uses on–off keying (OOK) modulation, being in accordance with the specifications of the IEEE 802.15.7 standard. Next, in order to prevent any flickering, the VLC emitter must use a run-length-limited (RLL) code that guarantees that logic level “1” and level logic “0” have the same light intensity. In this case, due to previously demonstrated performances, the classical Manchester code was used. In terms of data rate, the current version of the in-vehicle VLC receiver enables data rates up to 250 kb/s. Nevertheless, megabits-per-second data rates will soon be available. The data frame begins with a synchronization frame that informs the VLC receiver that a new message is being received. Next, the message header provides the VLC receiver with information concerning the modulation technique, the coding technique, the data rate, and the length of the data message. Then, the frame contains the data to be transmitted, followed by a stop byte that informs the VLC receiver that the message has come to the end. Last, a short inter-frame space is integrated to separate neighboring frames. The microcontroller board can generate its data to be transmitted or it can be interfaced with a different device that provides the data to be transmitted through I2C, SPI, or CAN interfaces. Next, once the data frame is ready to send, the microcontroller controls the LED’s light intensity through an LED driver. Thus, a light beam containing the data is generated. From this point, the light is introduced into the optical fibers, where it travels throughout the fibers until it reaches the vehicle rooftop, providing in-vehicle ambient lighting and also wireless data transfer. Next, the light leaves the optical fibers and travels through the free space optical channel carrying the information toward the VLC receiver. Figure 4 shows the practical implementation of the in-vehicle VLC emitter, showing the intermediate implementation steps and also the final in-vehicle ambient lighting system with data transmission capabilities, whereas Table 1 summarizes the VLC emitter parameters.

### 2.3. Hardware Design and Implementation of the In-Vehicle Visible Light Communications Receiver

The schematic of the proposed VLC receiver is illustrated in Figure 3. The proposed mobile VLC receiver was developed based on a PIN photodiode optical detector. Compared to other photosensitive devices, PIN photodiodes have the fastest response times, providing support for higher data rates. For design simplicity and decent performances, a PDA100A2 amplified detector was chosen. This optical receiver has an 11 MHz bandwidth and a relatively large optical collecting area of 75.4 mm^2^ enabling a decent SNR.

**Figure 5:**
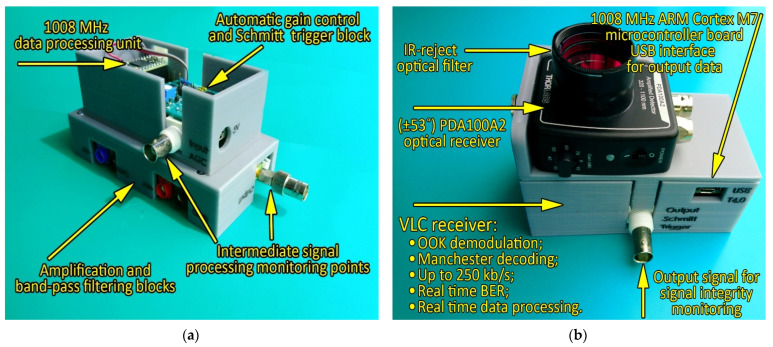
Visible light communications receiver prototype: (**a**) lateral view before integrating the optical receiver; (**b**) complete VLC receiver prototype.

Compared to outdoor VLC applications where the communication channel is significantly affected by the presence of numerous parasitic light sources (i.e., natural and artificial), the in-vehicle VLC channel is less exposed to intensive optical noise sources. Consequently, a wide FOV was considered for the VLC receiver. The wider FOV provides better compliance with user mobility within the vehicle and provides better connectivity at the cost of a lower SNR (when the VLC receiver is exposed to optical noise sources). To reduce the negative effect of potential optical noise sources and to enhance the SNR, an IR reject optical filter was integrated. This filter eliminates the sunlight spectral component outside the visible light spectrum (i.e., higher than 780 nm). For improved SNR performances, a narrow-band optical filter can be used as well. The optical receiver outputs a signal that is directly proportional to the incident optical light. As the output parameters of the VLC emitter are established based on the user’s requirements and preferences and not on the VLC requirements, the signal provided by the optical receiver might have relatively low values. Consequently, several amplification blocks were introduced, and in order to enable proper signal decoding, a band-pass filter was also integrated. In accordance with the Manchester coding power spectral density distribution, the band-pass of the filter was selected between 1 kHz and 1 MHz, enabling an adequate reception of Manchester encoded signals having a 250 kb/s data rate. Next, in order to be able to extract data when the value of the input signal is varying, an automatic gain control circuit (AGC) was also introduced. This circuit provides additional gain for signals that are too low, while it does not affect signals of proper amplitude (i.e., 1–3.3 V). The stabilized output of the AGC circuit is then supplied to the Schmitt trigger circuit, which reestablishes the square signal shape. Next, the signal is provided to the ARM Cortex M7 1008 MHz microcontroller, which processes the data in real time. Additionally, the microcontroller can also perform data analysis and provide the BER for qualitative link evaluation purposes. For improved clarity, the parameters of the VLC receiver are summarized in Table 2, and the final VLC receiver prototype is illustrated in Figure 5.

## 3. Experimental Evaluation of the In-Vehicle Visible Light Communications Data Transmission System

This section debates the aspects related to the experimental investigation of the in-vehicle VLC prototype and of the newly introduced in-vehicle VLC concept. It also provides evidence concerning the viability of the proposed concept in which VLC technology is used with light being distributed by optical fibers through the ceiling of the car.

### 3.1. Coupling Efficiency Evaluation

As presented in the previous sections, a unique feature of the proposed concept comes from the fact that, unlike previous VLC systems, this one uses optical fibers to distribute the light. Nevertheless, it is well known from previous experience in fiber optic communications that the coupling between LEDs and optical fibers leads to important coupling losses. In optical fiber communications, this issue contributes to a reduced communication distance which slowly led to the replacement of the LEDs with laser diodes. Nevertheless, in this case, fiber optics are mainly used for decorative and aesthetical purposes as the main function, and thus, the reduced coupling efficiency does not represent a serious issue.

In order to have an idea on this topic, light irradiance was measured at the LED output and also at the optical fiber output. As expected and as shown in Table 3, the irradiance at the output of the 500 optical fibers is significantly smaller compared to the one at the LEDs output, having a reduction of total power emission to around 12%. Nevertheless, from an energetic point of view, the 3 W consumption of the RGB LED is insignificant with respect to a vehicle’s electric consumption. On the other hand, the reduced optical power will most probably lead to a rather low SNR at the VLC receiver level.

### 3.2. Signal-to-Noise Ratio Analysis

**Figure 6:**
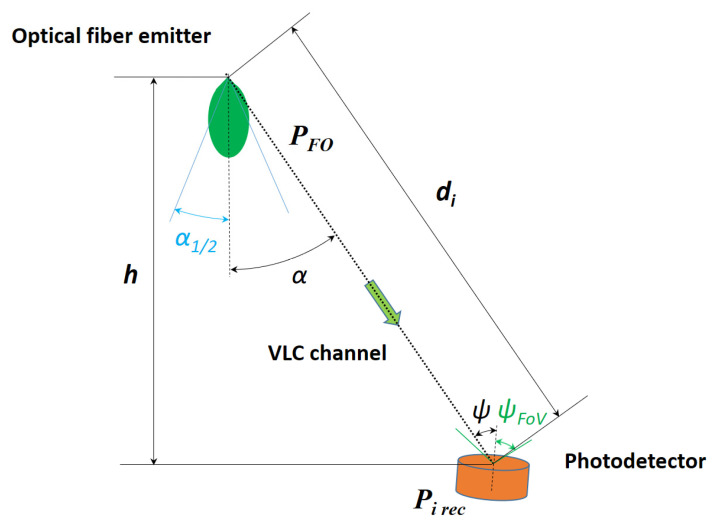
Schematic for the DC-gain estimation for VLC channel.

Based on the irradiance measurement performed in Section 3.1, this section aims to provide an analysis focused on the SNR distribution within the vehicle. In order to estimate the SNR values in a passenger car, the DC-gain of the VLC channel will be first calculated based on the parameters illustrated in Figure 6, considering for the beginning one fiber optic as a source of light, and starting with the following relation for $0<\psi <\psi_{FoV}$ [33,43]:

(1) $$H\left(0\right)=\frac{A}{d^{\gamma }}R_{0}\left(\alpha \right)T_{s}\left(\psi \right)g\left(\psi \right)\cos\psi \;$$

where *A* is the sensitive area of the PIN photodiode, $R_{0}\left(\alpha \right)$ is the emitter’s radiant intensity, $T_{s}\left(\psi \right)$ is the optical filter’s transmission factor, *g*(*ψ*) is the optical gain obtained when collimation lenses are used, and the γ exponent determines the power loss with the distance. It should be noted that *H*(0) *=* 0 whenever $\psi \geq \psi_{FoV}$. Because the radiant intensity can be considered a generalized Lambertian transmitter, it can be determined based on the relation:

(2) $$R_{0}\left(\alpha \right)=[\left(m+1\right)/2\pi ]\cos^{m}\alpha \;$$

where the order *m* for radiation directivity is established with the help of α12, the semi-angle at half power:

(3) m=−ln2ln(cosα12) 

In this experiment a collimator was not used, so the optical gain will be ignored. The IR reject filter will be considered omnidirectional, so the transmission factor can be approximated with a constant. The power loss of light in the air is proportional to the square of the distance between the transmitter and receiver, so γ=2. In addition, the orientation of the receiver can be approximated as facing the rooftop, therefore the incidence angle will be considered α=ψ, and cosα=h/d.

In the end, the DC-gain for the VLC channel in this simulation for $0<\alpha =\psi <\psi_{FoV}$ will be dependent only by the distance between the photodetector and the *i*th fiber optic emitter for a determined height:

(4) $$H_{i}\left(0\right)=\frac{AT_{s}\left(m+1\right)h^{m+1}}{2\pi d_{i}^{m+3}}$$

The power received from the *i*th fiber optic source of light, having the transmitted power $P_{FO}$, can be calculated with the relation (5):

(5) $$P_{i\;rec}=H\left(0\right)P_{FO}$$

so, in order to have the total received power $P_{r}$ for a determined position of the photodetector, the individual power from all 500 optical fibers must be considered:

(6) $$P_{r}=\overset{500}{\underset{i=1}{\sum }}P_{i\;rec}=\overset{500}{\underset{i=1}{\sum }}\frac{AT_{s}\left(m+1\right)h^{m+1}}{2\pi d_{i}^{m+3}}P_{FO}$$

Having the total power received from all the individual optical fibers, the estimation of total received noises must be considered in order to determine the signal-to-noise ratio. Photodiode current is mainly affected by two types of noise. The first one is shot noise variance, mainly ambient-induced, caused by the discrete nature of light, and the second one is thermal noise variance:

(7) $$\sigma^{2}=\sigma_{shot}^{2}+\sigma_{thermal}^{2}$$

Finally, the signal-to-noise ratio can be modeled based on the formula:

(8) $$SNR\left[\mathrm{dB}\right]=10log\frac{{\left(RP_{r}\right)}^{2}}{\sigma^{2}}$$

where *R* is the photodiode’s responsivity.

The simulation parameters are detailed in Table 4.

**Figure 7:**
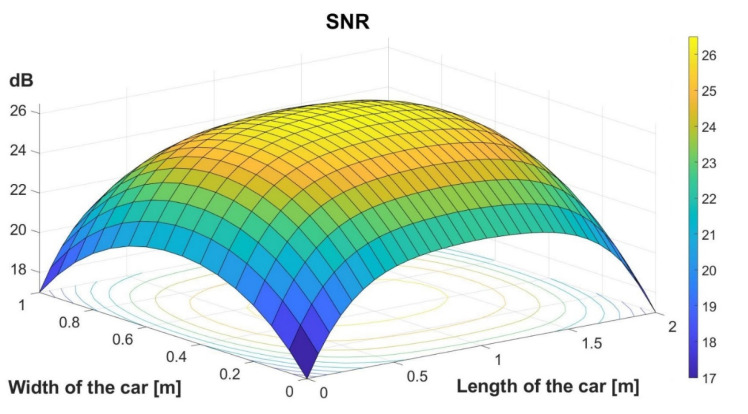
SNR distribution.

Figure 7 shows the SNR distribution modeled in the car. As one can see, the estimated SNR has very good values, between 17 and 26 dB, so the expectation in this scenario is to have an extremely low BER value for the entire covered area.

### 3.3. VLC Emitter Spectral Analysis and Multiple Input Multiple Output Perspectives Evaluation

**Figure 8:**
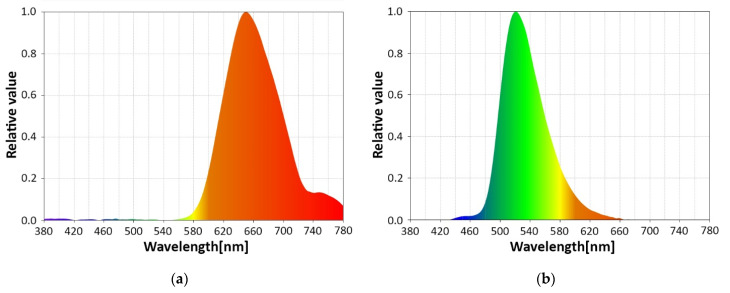

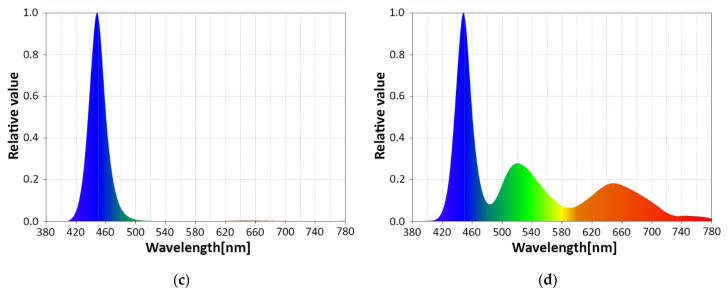
Spectral analyses of: (**a**) VLC emitter red light; (**b**) VLC emitter green light; (**c**) VLC emitter blue light; (**d**) VLC emitter white light.

As mentioned in Section 2.2, the VLC emitter is englobing a 3 W RGB LED that is used to enable the passenger of the car to personalize the ambient lighting. The RGB LED facilitates users to select one of the RGB colors, an intermediate color established by balancing the proportions of the RGB LEDs, and also the white color. Figure 8a–d presents the spectral analyses for the three RGB colors, and also the one of the white light, which will be used in the next part of the experimental tests. One can see that the LEDs have a rather wide spectral bandwidth, but even so, the three RGB colors can be easily distinguished, enabling in turn the possibility to use wavelength division multiplexing (WDM) for enhanced data rates using multiple input multiple output (MIMO) approach, or for compatibility with multiple applications.

**Figure 9:**
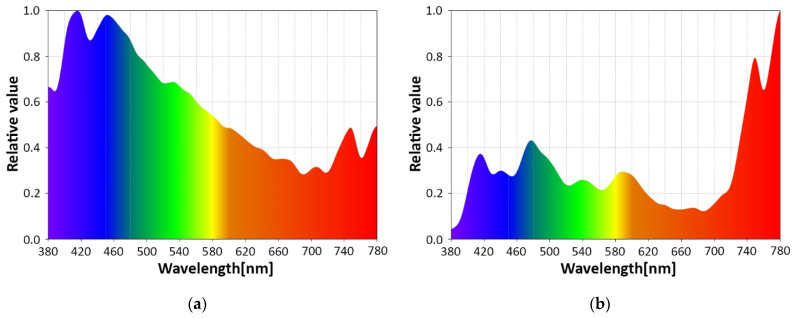
Spectral analyses of the VLC channel: (**a**) outdoor afternoon conditions; (**b**). in-vehicle conditions.

Another aspect that is revealed by the spectral analyses shown in Figure 9 is related to the effect of the sunlight at the VLC receiver level and also to the effect of the vehicle’s tinted windows on the SNR level. As is well known, sunlight has a different spectral distribution with respect to the season, moment of the day, and presence or absence of clouds. Figure 9a shows the spectral analysis at the moment these experiments were performed, and Figure 9b shows the spectral analysis inside the vehicle. As one can see, the vehicle’s window treatment acts as an optical filter, significantly reducing the influence of the optical spectral components whose wavelengths are lower than 620–640 nm, improving the SNR at the VLC receiver level.

### 3.4. Data Communication Performance Evaluation

**Figure 10:**
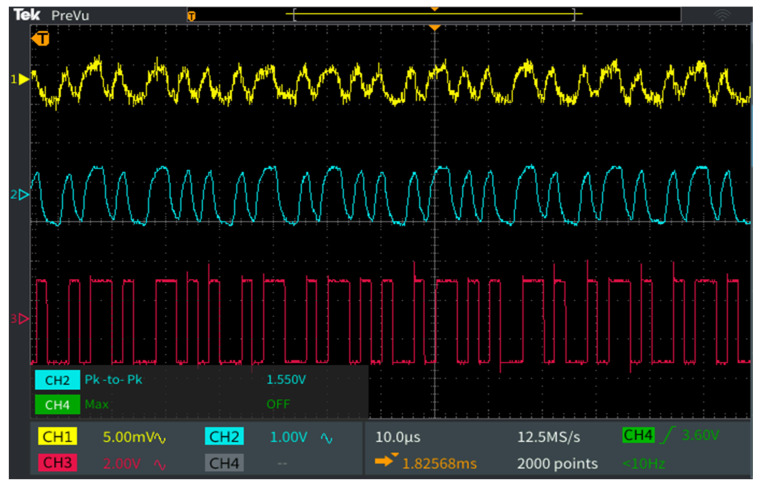
Oscilloscope screen showing the signal processing plan at the VLC receiver level: Channel 1 (yellow) shows the output of the transimpedance amplifier adjusted at 10 dB gain; Channel 2 (cyan) provides the output of the amplified signal, after 1 kHz–1 MHz band-pass filter that will be feed into the Schmitt trigger circuit; Channel 3 (magenta) displays the final data signal that will be provided to the microcontroller for the data extraction process.

**Figure 11:**
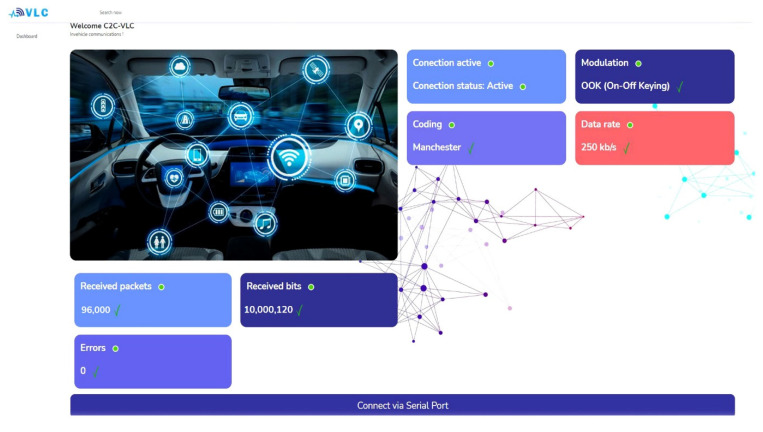
Graphical user interface displaying the parameters of the communication.

After evaluating the coupling efficiency and the multiplexing potential, this section has the purpose to evaluate the in-vehicle VLC prototype data communication performances. For this aim, the VLC emitter transmits a 10 million bit sequence by repeating a predefined message of 104 bits. The VLC photodetector receives the incoming light, transforms it into an electrical signal, processes the signal until the initial square shape is restored and the amplitude reaches a 3.3 V level that can be processed by the microcontroller, and then feeds the signal to the 1008 MHz data processing unit. Next, the microcontroller synchronizes with the incoming signal, processes the header data, and extracts information concerning the modulation, coding, data rate, and message length, and then, based on this information, it extracts the binary data. Then, in order to have feedback concerning the quality of the link, the microcontroller performs a real-time BER analysis, by comparing the received bits with the ones it is supposed to receive and which are stored in the memory. Alternatively, when not performing BER analysis, the VLC receiver can feed the data to a device that uses it for various applications (entertainment applications such as media players or the internet). Figure 10 shows the data reception process at the VLC receiver level, and Figure 11 shows the GUI with the link evaluation parameters. Table 5 shows the BER performance for various testing conditions. The experimental results show that in limited parasitic lighting conditions the proposed system is able to maintain a BER lower than 10^−7^, as was expected from the simulation results, for distances between 10 cm and up to 80 cm (the typical distance between the car ceiling and the car seat). It should be mentioned here that for higher results confidence, each experiment was repeated three times. The experimental evaluation also showed that in the absence of intense optical noise sources, the VLC receiver is able to process optical signals that have an irradiance value around 1 µW/cm^2^ at the receiver level, and the signal processing part is able to process signals that have an initial value as low as 3–4 mV. The experimental results also revealed that the uniform distribution of the 500 optical fibers enables constant performance within the car. This affirmation is also supported by the simulation results presented in Section 3.2. Thus, as shown in Figure 7, the VLC receiver experiences SNR levels between 17 and 26 dB. In these circumstances, although the SNR level is lower in certain points, it is sufficiently high not to influence the BER results in the 10^−7^ domain, although lower BER could be expected in certain conditions where direct sunlight is reaching the VLC receiver’s photosensitive element.

In terms of data rate, the system’s performance is limited to 250 kb/s due to the 1 MHz bandwidth limitation of the implemented prototype. Nevertheless, the low SNR definitely enables higher data rates. However, as the purpose of this article is to demonstrate a new VLC technology use case rather than to establish record communication data rates, higher data rates were not targeted.

## 4. Debate on the Experimental Results and Discussion about This Work

### 4.1. Debate on the Experimental Results, and Positioning of This Work with Respect to the Current State-of-the-Art in the Visible Light Communications Area

The experimental results have shown that the proposed in-vehicle data communications prototype is able to provide in-vehicle users’ connectivity, for passengers located in different places in the car. In this case, the uniform spreading of the optical fibers distributing the light and the wide VLC detector reception angle enabled an extended coverage area within the car. In terms of communication range, the system demonstrated a reliable distance that reached up to 80 cm, covering the needs of in-vehicle applications and enabling connectivity for all users within the vehicle. This is the typical distance between a standard vehicle rooftop and the user seat. The experimental results have demonstrated a 250 kb/s data rate and a BER lower than 10^−7^. Even though 250 kb/s is not impressive compared to some of the best-performing VLC systems, it must be emphasized that the main purpose of this work was to propose and to demonstrate a new VLC use-case scenario, rather than to establish a record in data rate communication. Additionally, preliminary experimental results have shown that the data rate can be further increased, and higher data rates can be achieved with the help of parallel transmission using the three RGB wavelengths in a MIMO setting. In addition, by using the WDM concept, the system could be easily adapted to provide simultaneous multiple users support. Consequently, based on the experimental results, one can see that the proposed prototype is able to provide in-vehicle wireless data communications, ensuring adequate connectivity, good BER performances, and moderate data rates. Nevertheless, as this is the first version of the prototype, and the purpose of this work was mainly in providing a technology demonstrator rather than a final commercial system, additional steps are required until a final product is ready. In this case, the data rate needs to be further improved, bidirectional communication must be established, and miniaturization of the final product must be ensured. Further on, the data rate improvements could be achieved with the help of the WDM concept as mentioned above and shown in Section 3.3, but also with the help of superior modulation techniques such as orthogonal frequency division multiplexing (OFDM) [44,45] or different multilevel modulations. On the other hand, a solution for bidirectional communications could be an optical receiver fixture on the vehicle’s rooftop for an Infrared (IR) upload function. Thus, based on the current development of VLC technology, the proposed concept can be further improved in order to become a commercial product.

As shown in Section 1, VLC technology has numerous applications in various areas. These areas mainly include indoor applications, industrial applications, underwater applications, and automotive applications. In the various indoor applications, the main purposes were to improve the data rates, and thus, gigabits per second data rates have been demonstrated in laboratory conditions [10,11], and on the other hand, to develop mechanisms that enable multiple users’ coexistence and inter-cell handover [46]. The area of automotive applications is also a very active VLC research area. In this domain, the work was focused on developing vehicle-to-vehicle, vehicle-to-infrastructure, and infrastructure-to-vehicle VLC systems, focusing on improved robustness and noise resilience, medium to long communication ranges, and low latencies [29,30,31,32,33,34,35]. The use of the VLC in the automotive area has the potential to significantly improve road traffic safety and also to generate important investments and progress, considering the importance of the automotive economic sector. Additionally, developing new automotive VLC applications is essential, facilitating the popularization of VLC technology to the general public. In this context, the concept introduced by this article is innovative and it is envisioned for a domain that can generate important benefits for car manufacturers but also for car users.

### 4.2. Debate on the Importance of this Work and on the Future Use of Visible Light Technology in In-Vehicle Data Transmission Applications

The main contribution of this article comes from the fact that it has proposed and demonstrated a new VLC technology use case. As far as we know, the current VLC literature does not mention the use of VLC technology for in-vehicle data transmission purposes. Additionally, different from many of the works that propose a new application area for a technology, in this article, a prototype was developed, implemented, and tested. The experimental evaluation of the in-vehicle data transmission prototype has demonstrated the viability of the proposed concept. Additionally, the experimental evaluation also showed that although the performances of the proposed prototype are limited at the moment compared to the ones achieved in other VLC applications, there is potential for significant development and to achieve similar performances. Nevertheless, as this is the first step from a long line, additional work efforts are still required. In the near future, as this in-vehicle system will be further developed and the VLC receiver will get more compact, many applications can be implemented. Such applications may include in-car video streaming, in-car audio streaming, personalized passenger assistance services, or in-vehicle LiFi.

Another important aspect related to this work is that in this case instead of distributing the light using a high number of LEDs, a single RGB LED was used and the light was distributed through the rooftop of the car using 500 optical fibers. Thus, this approach has the potential to enable a significantly lower cost, but also to demonstrate a new utilization of the VLC and fiber optics technologies. Here, from an economic perspective, the cost of optical fibers is much lower than the cost of LEDs. On the other hand, this approach has as a possible disadvantage rather low coupling efficiency between LEDs and optical fibers. This means that a significant part of the light produced by LEDs is lost (as revealed in Section 3.1), but this is insignificant with respect to a car’s energy consumption. Additionally, it should be remembered that the integration of the optical fibers had an initial purpose as ambient light, and that the data transmission was integrated as a supplementary function. Thus, one can see that the energy loss associated with the low coupling efficiency is mainly justified by the user’s preferences and not by the data broadcasting function.

## 5. Conclusions

After a period of uncertainty, the feasibility of visible light communications has been gradually demonstrated. Nowadays, research in this area is focused on continuously improving technology’s performances in terms of achievable data rates, resilience to optical noise, user mobility, and multiple users’ coexistence. Then again, the research community is also concentrated on identifying more VLC applications and on convincing the wide public to embrace this relatively new technology.

In light of the above-mentioned, this article has introduced a new VLC use case, namely, the in-vehicle VLC utilization. Thus, in order to demonstrate the applicability of this new utilization scenario, a new VLC prototype was implemented and experimentally evaluated. Unlike in previous cases, the VLC emitter was developed based on a tunable in-vehicle ambient lighting system. On the receiver side, a new VLC receiver prototype was tested. The intensive experimental results have shown that the proposed VLC system is able to provide continuous data transfer for the occupants of a car. In terms of performance, the experimental results showed a BER lower than 10^−7^ for a 250 kb/s data rate. Additionally, preliminary results confirmed the high compatibility with MIMO applications, enabling in turn higher data rates and/or multiple users’ coexistence. Future work on this topic will focus on demonstrating the tens of megabits per second data rates and also on continuing the exploitation of the MIMO possibilities.

## Figures and Tables

**Table 1:** Visible light communications emitter parameters.

VLC Emitter Parameter	Feature/Measure
VLC emitter	In-vehicle ambient lighting system based on: - 3 W RGB LED - 500 fiber optics that distribute the light within the vehicle
Optical irradiance measured at the output of an optical fiber	120 µW/cm^2^ at 1 cm distance
Semi-angle emission at half power at the output of an optical fiber	±30°
VLC emitter data processing unit	1008 MHz ARM Cortex M7 microcontroller (overclocked 680 MHz version)
VLC emitter modulation/decoding/data rate capabilities	OOK/Manchester/250 kb/s

**Table 2:** Visible light communications receiver parameters.

VLC Receiver Parameter	Feature/Measure
VLC photodetector type	PIN photodiode-based PDA100A2 optical detector
Optical filter characteristics	IR reject optical filter (eliminates spectral components higher than 780 nm)
Field of view	±53°
Bandwidth	1 MHz
Data processing unit	1008 MHz ARM Cortex M7 microcontroller (overclocked 680 MHz version)
Demodulation/decoding/data rate capabilities	OOK/Manchester/11–250 kb/s
VLC receiver capabilities	Real-time data processing of data rates up to 250 kb/s and real-time bit error ratio processing

**Table 3:** Summary of the coupling efficiency experimental results.

Parameter	Value
LEDs irradiance at 5 cm distance (µW/cm^2^)	20,100
Irradiance at 5 cm distance from the optical fiber output (µW/cm^2^)	4.8
Distributed irradiance for 500 optical fibers (µW/cm^2^)	2400
Coupling and transmitted efficiency (%)	12

**Table 4:** Simulation parameters for the in-vehicle SNR distribution modeling.

Parameter	Description	Value
*P_FO_*	Optical fiber output power	260 μW
*N*	Number of optical fibers	500
*T_s_*	Transmission factor of the IR reject optical filter	1
*A*	Active area of the photodetector	75.4 mm^2^
*ψ*	Angle of incidence	0–90°
*ψ_FoV_*	FoV of the receptor	±53°
*α*	Angle at the emission	0–90°
*α*_1/2_	Semi-angle at half power	±30°
*h*	Rooftop receptor distance	<0.80 m
*γ*	Path loss exponent	2
*R*	Photodetector’s responsivity	0.45 A/W
*BW*	TIA bandwidth—10 dB	1.4 MHz
*NEP*	Noise equivalent power	6.75 × 10^−12^ W/Hz^1/2^
*L*	Length of the car interior	2 m
*w*	Width of the car interior	1 m
*NoiseTIA*	TIA noise—10 dB	195 μV
*Irrad*	Solar irradiance inside the car	50 μW/cm^2^

**Table 5:** Summary of the experimental results for the proposed setup.

Parameter of the VLC System	Value
Modulation	OOK
Coding	Manchester
Data rate (kb/s)	250
VLC Distance (cm)	10–80
BER	<10^−7^
Confidence level	95%

## Data Availability

Not applicable.
